# Spinal cord hypermetabolism extends to skeletal muscle in amyotrophic lateral sclerosis: a computational approach to [18F]-fluorodeoxyglucose PET/CT images

**DOI:** 10.1186/s13550-020-0607-5

**Published:** 2020-03-23

**Authors:** Matteo Bauckneht, Rita Lai, Alberto Miceli, Daniela Schenone, Vanessa Cossu, Maria Isabella Donegani, Stefano Raffa, Anna Borra, Stefano Marra, Cristina Campi, Annamaria Orengo, Anna Maria Massone, Alberto Tagliafico, Claudia Caponnetto, Corrado Cabona, Angelina Cistaro, Adriano Chiò, Silvia Morbelli, Flavio Nobili, Gianmario Sambuceti, Michele Piana, Cecilia Marini

**Affiliations:** 1 Nuclear Medicine Unit, IRCCS Ospedale Policlinico San Martino, Genoa, Italy; 2 Department of Mathematics (DIMA), University of Genoa, Genoa, Italy; 3 Department of Health Sciences (DISSAL), University of Genoa, Genoa, Italy; 4 Department of Medicine-DIMED, Padova University Hospital, Padua, Italy; 5 Neurology Clinic, IRCCS Ospedale Policlinico San Martino, Genoa, Italy; 6 Department of Neuroscience, Rehabilitation, Ophthalmology, Genetics, Maternal and Child Health, University of Genoa, Genoa, Italy; 7 Nuclear Medicine, E.O. Ospedali Galliera, Genoa, Italy; 8 ALS Center, Rita Levi Montalcini Department of Neuroscience, University of Turin, Turin, Italy; 9 AUO Città della Salute e della Scienza, Turin, Italy; 10 CNR Institute of Molecular Bioimaging and Physiology (IBFM), Segrate (MI), Italy

**Keywords:** Amyotrophic lateral sclerosis, Skeletal muscle, FDG, Prognosis

## Abstract

**Purpose:**

Amyotrophic lateral sclerosis (ALS) is a neurodegenerative disease leading to neuromuscular palsy and death. We propose a computational approach to [18F]-fluorodeoxyglucose (FDG) PET/CT images to analyze the structure and metabolic pattern of skeletal muscle in ALS and its relationship with disease aggressiveness.

**Materials and methods:**

A computational 3D method was used to extract whole psoas muscle’s volumes and average attenuation coefficient (AAC) from CT images obtained by FDG PET/CT performed in 62 ALS patients and healthy controls. Psoas average standardized uptake value (normalized on the liver, N-SUV) and its distribution heterogeneity (defined as N-SUV variation coefficient, VC-SUV) were also extracted. Spinal cord and brain motor cortex FDG uptake were also estimated.

**Results:**

As previously described, FDG uptake was significantly higher in the spinal cord and lower in the brain motor cortex, in ALS compared to controls. While psoas AAC was similar in patients and controls, in ALS a significant reduction in psoas volume (3.6 ± 1.02 vs 4.12 ± 1.33 mL/kg; *p* < 0.01) and increase in psoas N-SUV (0.45 ± 0.19 vs 0.29 ± 0.09; *p* < 0.001) were observed. Higher heterogeneity of psoas FDG uptake was also documented in ALS (VC-SUV 8 ± 4%, vs 5 ± 2%, respectively, *p* < 0.001) and significantly predicted overall survival at Kaplan–Meier analysis. VC-SUV prognostic power was confirmed by univariate analysis, while the multivariate Cox regression model identified the spinal cord metabolic activation as the only independent prognostic biomarker.

**Conclusion:**

The present data suggest the existence of a common mechanism contributing to disease progression through the metabolic impairment of both second motor neuron and its effector.

## Introduction

Amyotrophic lateral sclerosis (ALS) is a fatal neurodegenerative disease of adult life, characterized by a progressive impairment of motor function. Its time course is extremely variable, with time elapsing from diagnosis to death or severe inability ranging from months to years. This heterogeneity prevents an accurate outcome prediction and hampers the development of new therapeutic approaches. However, the lack of validated biomarkers and the limited knowledge about disease mechanisms inevitably hampers the identification of target patients and limit a correct identification of treatment effects. This uncertainty thus raises an urgent need to develop biomarkers able to characterize the mechanisms underlying disease progression.

PET/CT imaging potentially allows to integrate information derived from all ALS target tissues, in particular from the brain, spinal cord, and skeletal muscles in living patients, targeting a variety of potential pathophysiological mechanisms related to tissue metabolism, inflammation, and oxidative stress. We recently reported the potential of a computational approach in extracting spinal cord metabolism from FDG PET/CT scanning in ALS patients [1]. This approach documented a metabolic activation of this nervous site facing an opposite pattern in the brain cortex that showed a generalized reduction in tracer uptake [2].

In the present study, we aimed to integrate our previous observation with the evaluation of structural and metabolic features of psoas muscles. This region was selected because it is always included in whole-body PET/CT scans and is less influenced by voluntary activity during the FDG uptake phase. This analysis was complemented with the evaluation of myocardial tracer retention, since the comparison with this non-voluntary striated muscle allowed us to verify whether the metabolic activation selectively affects the motor chain or, rather, it reflects a systemic phenomenon involving all striated muscles regardless their connection to the lower motor neuron.

## Material and methods

### Patients with amyotrophic lateral sclerosis

The study included 62 patients retrospectively recruited from our published database with definite, probable, or probable laboratory-supported diagnosis of spinal-onset ALS according to the revised El-Escorial criteria [3]. None of the enrolled patients had any history of other neurological disorders, cerebrovascular disease, diabetes mellitus, or systemic inflammatory disease. All subjects provided signed informed consent to enter the study that was approved by the Ethics Committees of IRCCS Ospedale Policlinico San Martino in Genoa and of AUO Città della Salute e della Scienza in Turin, Italy.

As part of our clinical procedure, patients were submitted to our follow-up program by medical examination or phone interview. Over the follow-up period of 60 months data were available for 56/62 patients while no information could be obtained for the remaining six.

### Control subjects

Findings obtained in ALS patients were compared with the corresponding data in control subjects selected from two different databases. Metabolic activity and structure of spinal cord as well as of psoas muscle were compared with the corresponding findings in 62 subjects selected submitted to FDG PET/CT scan > 1 year after complete removal of histologically diagnosed melanoma, subsequent histologically negative sentinel lymph node, and no evidence of relapse at least 2 years after surgery [4]. These records were extracted from the databases of the two centers and selection of each subject was performed to optimize the case-control criterion according to the used scanner, sex, and age.

For brain analysis, FDG uptake of ALS patients was compared with the published corresponding data in 44 normal volunteers with normal findings at neuropsychological evaluation and brain MRI as previously defined [5].

### PET/CT imaging

All PET/CT scans were acquired according to current guidelines [6, 7]. All subjects were studied in the early morning after fasting for 12 h. Serum glucose was assessed, and an antecubital vein was cannulated. Patients were invited to lie for 20 min in a silent and darkened room, with eyes closed and ears unplugged. FDG (4.8-5.2 MBq/kg body weight) was then injected 45–60 min before 3D scan using an integrated PET/CT scanner (Biograph 16-Hirez, Siemens or Discovery ST-E System, GE Healthcare). In all cases, the 15-min cerebral acquisition was followed by whole-body imaging in arms down position.

In both centers, PET data were reconstructed into a 128 × 128 matrix using a 3D iterative reconstruction algorithm (OSEM, three iterative steps, eight subsets). Raw images were scatter-corrected and processed using a 3D Gaussian filter, while CT was used for attenuation correction.

Image quality control documented a spatial resolution of 4.0 mm full width at half-maximum for both scanners. According to standard procedures of both labs, the two imaging systems were cross calibrated using a cylinder of 20 cm diameter and 20 cm length filled with a solution containing 100 MBq of ^68^Ge. Images were reconstructed with the same algorithm used for the clinical protocol [6, 7]. Finally, the entire CT dataset was co-registered with the 3D PET images using commercially available software interfaces.

### Whole-body FDG-PET/CT analysis

Muscular FDG uptake was analyzed in both psoas muscles. This site was selected because its contractile activity is minimized with patient resting in the supine position and thus in the interval between FDG injection and PET/CT acquisition; moreover, a large part of its volume is systematically included in a whole-body PET/CT acquisition and, finally, its size and structure have been proposed as relevant prognostic predictors in different disease states [8, 9].

Usually, psoas muscles are evaluated at CT by selecting a single muscle slice at the level of the third lumbar vertebra. To improve the accuracy of this evaluation, we developed a computational approach as to extract the entire recognizable muscle volume. The algorithm follows a slight modification of the general strategy previously validated by our lab for the assessment of bone marrow metabolic activity [4, 10]. According to this approach, the first step implies a visual inspection of CT images to define the proximal insertion of both psoas muscles at starting from the soma of D12 vertebra. To standardize volume definition, the caudal limit of the investigated volume was set at the plane crossing L5-S1 junction. Included slices were fed into an in-house developed software that utilizes histogram equalization and edge detection in order to segment the psoas volume. In the case of not-closed, not-connected edges, the software applies an α-shape algorithm [11] to identify the region corresponding to the inner muscle. After this automatic recognition, each slice was used to construct a binary mask, with the value set at 1 inside the domain representing the muscle and 0 elsewhere. The mask was adjusted in order to account for the differences between CT and PET pixel dimensions, downsampling the CT masks to the PET resolution. The post-processed masks were eventually multiplied against the PET data to extract the information on the FDG uptake in correspondence of the muscle voxels. The final product was thus two DICOM file series, reporting the CT and PET data, respectively. CT image was used to compute psoas volume and AAC (expressed in Hounsfield units). PET images were analyzed to estimate psoas FDG uptake, expressed as average standardized uptake value (SUV) and its heterogeneity expressed by the variation coefficient (VC-SUV, expressed in %), defined as the ratio between N-SUV SD and N-SUV average within the voxels of the two muscles of each patient. Spinal cord analysis was performed as previously described [1, 2]. Finally, myocardial FDG uptake was assessed as previously described [12]. Briefly, a volume of interest (average 6 ± 3 mL) was identified on the visible left ventricular (LV) myocardium on PET images while CT series was used as a reference, only in case of absent cardiac uptake. The myocardial volume of interest was set at a minimum value of at least 10 mL. The average SUV in this volume was estimated and divided for the corresponding average value in the liver to obtain myocardial N-SUV.

According to our procedure, all SUVs were divided by the corresponding average liver SUVs to account for possible differences in scanner sensitivity as to obtain the normalized SUVs (N-SUVs). In order to account for the obvious effect of body conformation and gender, psoas volume was normalized for the expected body volume calculated by the estimation of ideal body weight (IBW) according to the conventional formula of Robinson et al. [13].

### Brain FDG-PET/CT analysis

Original DICOM data of brain acquisition were converted to NifTI-1 format using SPM8 DICOM Import [14]. PET images were normalized to a customized previously published template [15] and smoothed with an 8-mm full width at half maximum Gaussian Kernel. Brain Map Ginger ALE 2.3 (Eickhoff SB, Laird AR) was used to convert coordinates of significant clusters in the Montreal Neurological Institute (MNI) space into Talairach coordinates. Brodmann areas (BAs) were then identified at a range of 0 to 3 mm from the corrected Talairach coordinates of the SPM output isocenters, after importing the corrected coordinates by means of Talairach client (http://www.talairach.org/index.html).

After this preliminary processing, the preprocessed NifTI-1 PET images were converted in whole-brain SUV parametric maps dividing the product between radiotracer concentration (kBq/ml) and body weight (in kg) by the injected FDG dose (in MBq) [16–18]. Thereafter, WFU PickAtlas and NiftyReg were used to automatically identify volumes of interest corresponding to the motor cortex (Brodmann Area 4 (BA4)) in both hemispheres. All healthy control subjects were submitted to exclusive brain PET imaging. Accordingly, due to the absence of liver FDG uptake in this cohort, motor cortex FDG accumulation was analyzed considering the raw SUV since normalization for liver uptake was not possible.

### Statistical analysis

All data are reported as means ± SD. Unpaired or paired *t* tests were used, as appropriate, to compare spinal cord N-SUV and motor cortex SUV, as well as all the computationally obtained FDG PET/CT variables describing psoas muscles (volume, AAC, N-SUV, VC-SUV). Linear regression analysis was performed using the least-squares method. A *p* value < 0.05 was considered significant. To assess the prognostic relevance of each of the following seven variables (i.e., age, ALS functional score and spinal cord N-SUV, psoas volume, N-SUV, VC-SUV and, finally, motor cortex SUV), the 56 patients were divided into two groups using the median value of that variable, thus resulting in two groups that differed in composition time by time according to the selected parameter. Survival was analyzed using the Kaplan–Meier method and compared using the log-rank test. Thereafter, a set of univariate and multivariate Cox proportional hazard models were fitted to the data. In the univariate analysis, the incidence of death was modeled as a function of each of the seven variables. Then, these same variables were tentatively included in a multivariate Cox model by means of a step-down (backward) procedure, based on the likelihood ratio test: variables with a *p* value > 0.1 were removed from the model. Proportionality assumptions were assessed as previously described [19].

## Results

### Clinical characteristics of the patient cohort

Within ALS cohort, there were 36 males and 26 females, mean age was 62 ± 12 years, body weight was 69 ± 13 kg. ALSFRS-R score, collected for each patient at imaging date, ranged from 20 to 46/48 (average 39 ± 5). Overall, 22 and 40 subjects for either cohort were acquired with Siemens Hirez or GE Discovery, respectively. The time elapsed from ALS onset and PET/CT scanning was 18 ± 14 months (range 3–82). For the 56 available patients, follow-up lasted 26 ± 14 months after imaging (median 22 months, range 2–58 months). During this period, 21/56 (37%) patients died, in most cases because of respiratory complications.

### PET/CT description of ALS effect on central nervous system

In agreement with our previous reports, the spinal cord and motor cortex displayed an opposite metabolic response to ALS. As shown in Table 1, the average FDG uptake of the whole spinal cord was significantly higher in patients than in controls. This difference was evident in cervical segments while it was not appreciable in the dorsal trait. This response of spinal cord metabolism was independent from demographic and clinical variables as well as from time elapsed from diagnosis to imaging. By contrast, the effect of ALS on tracer retention in the brain cortex was the opposite. Indeed, SUV in the brain cortex was lower in patients than in control subjects in both BA4 and whole brain (Table 1).

**Table 1:** FDG uptake data in ALS patients and control subjects

		Average	SD	Median	First quartile	Third quartile	IQR	*p* value
Spinal cord N-SUV	ALS patients	0.76	0.2	0.74	0.59	0.86	0.27	*p* < 0.05
	Control subjects	0.66	0.12	0.65	0.59	0.79	0.20	
Cervical spinal cord N-SUV	ALS patients	0.96	0.3	0.94	0.74	1.15	0.41	*p* < 0.01
	Control subjects	0.73	0.2	0.70	0.64	0.96	0.32	
Dorsal spinal cord N-SUV	ALS patients	0.69	0.22	0.65	0.53	0.79	0.26	*p* = ns
	Control subjects	0.64	0.11	0.66	0.25	0.40	0.15	
Brodmann Area 4	ALS patients	5.59	1.3	5.03	4.75	6.48	1.73	*p* < 0.01
	Control subjects	6.67	0.3	6.3	6.29	7.18	0.89	
Whole brain cortex	ALS patients	5.11	1.3	5.08	4.52	6.54	2.02	*p* < 0.01
	Control subjects	5.95	0.2	5.98	5.64	6.41	0.77	
Psoas muscle	ALS patients	0.45	0.19	0.43	0.36	0.55	0.19	*p* < 0.01
	Control subjects	0.29	0.09	0.28	0.23	0.35	0.12	

### PET/CT description of ALS effect on skeletal muscles

**Fig. 1:**
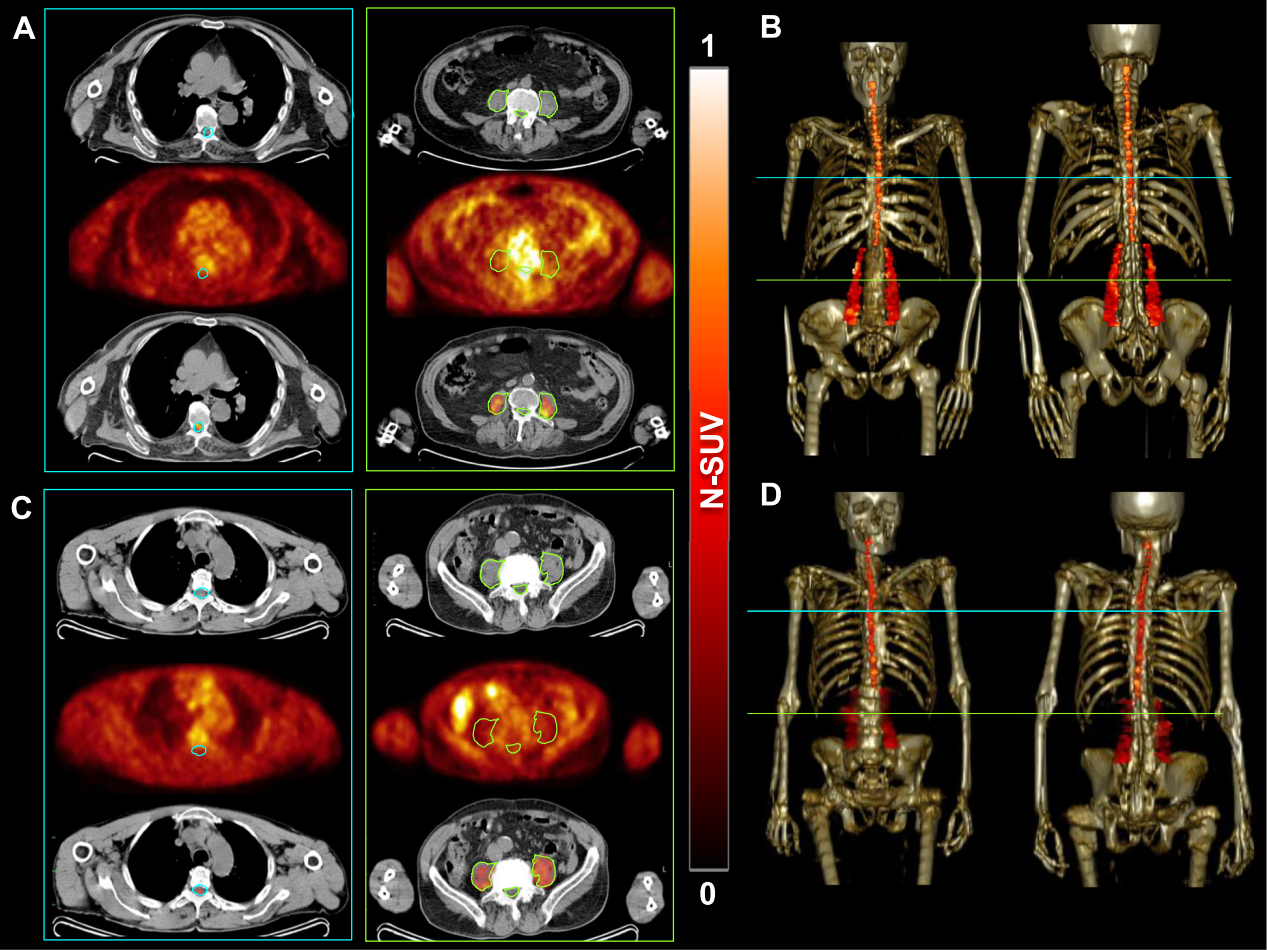
Examples of psoas muscle extraction from PET/CT images. Computational extraction of the spinal cord and psoas regions of interest from axial FDG PET/CT images in ALS patient (**a**) and healthy control (**c**). In panels **b** and **d**, the 3D reconstruction of obtained volumes of interest on the skeleton of these subjects are reported

An example of psoas muscle extraction from PET/CT images in reported in Fig. 1. ALS did not affect the CT indexes of psoas muscle composition. Indeed, AAC was remarkably similar in patients and controls (39.4 ± 8.4 vs 39.1 ± 11.3 HU, respectively, *p* = ns). By contrast, muscle mass was significantly affected by ALS, since volume normalized for IBW was significantly lower in patients compared to control subjects (3.6 ± 1.02 vs 4.2 ± 1.33 mL/kg, respectively, *p* < 0.01). Average FDG uptake in psoas muscles was significantly different between the two cohorts. Indeed, psoas muscle tracer retention was higher in ALS patients (average N-SUV 0.45 ± 0.19, median 0.43, IQR 0.19) compared to controls (average N-SUV 0.29 ± 0.09, median 0.28, IQR 0.12), respectively (*p* < 0.001). Moreover, ALS was also associated with increased heterogeneity of FDG accumulation, since the VC-SUV was higher in patients than in controls (VC-SUV 8 ± 4%, vs 5 ± 2%, respectively, *p* < 0.001). Of note, this difference did not involve the non-voluntary striated muscle represented by the myocardium, whose patients average N-SUV 1.52 ± 0.88, median 1.40 and IQR 2.12 were similar compared to controls (average N-SUV 1.73 ± 1.1, median 1.80, IQR 1.08), respectively, *p* = ns.

**Fig. 2:**
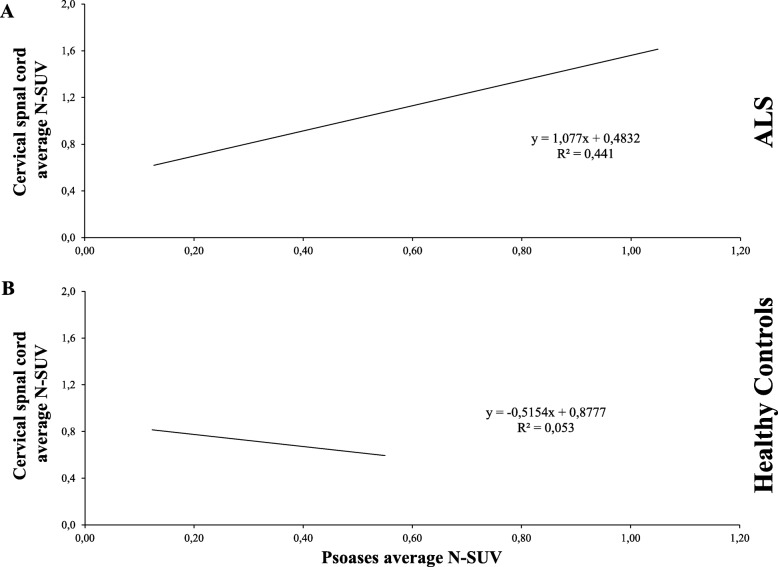
Psoas FDG uptake mirrors spinal cord hypermetabolism in ALS. In ALS, a direct correlation between the cervical spinal cord and psoas average N-SUV was documented (**a**), while these two indexes were largely independent in healthy controls (**b**)

ALS induced a similar response in skeletal muscles and spinal cord metabolic activity. Indeed, as shown in Fig. 2, psoas N-SUV was directly and significantly correlated with spinal cord N-SUV in patients but not in control subjects. By contrast, psoas muscle N-SUV was independent of the corresponding metabolic index in the whole brain and BA4 average SUV (Additional file 1: Figure S1).

### Metabolic pattern and patient outcome

**Fig. 3:**
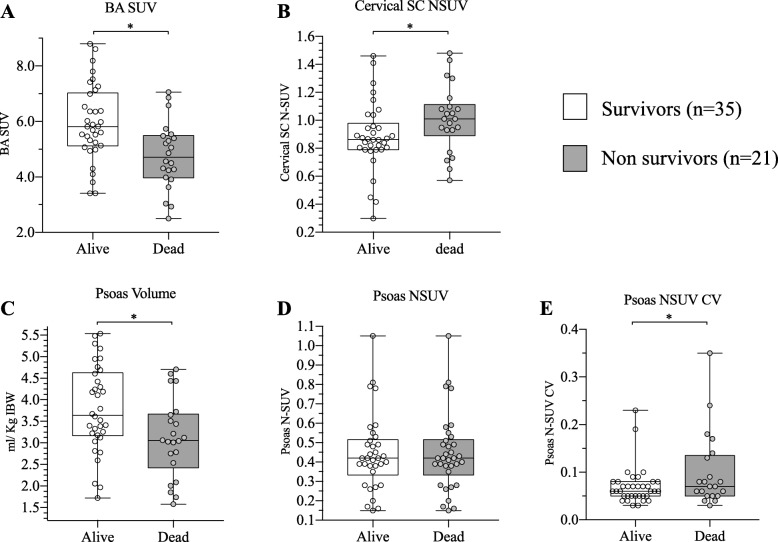
FDG uptake in BA4, spinal cord and psoas muscle in ALS according to patient outcome. Long-term survival was associated with a higher FDG accumulation in BA4 (**a**) and a lower FDG uptake in the cervical spinal cord (**b**). Psoas muscle showed a similar FDG uptake in the two subgroups (**d**) but greater homogeneity of tracer retention (**e**) and higher volumes (**c**) in survivors. *p < 0.05 vs controls

As a first step to evaluate their prognostic relevance, we first verified the presence of significant differences in measured indexes between the 21 patients who died during the clinical follow-up compared to the 35 survivors. As shown in Fig. 3, this analysis showed that higher mortality rates were associated with a higher FDG uptake in the spinal cord and a lower FDG accumulation in BA4. Psoas muscle showed a similar metabolic index in the two groups but higher volumes and greater homogeneity of tracer retention in survivors.

**Fig. 4:**
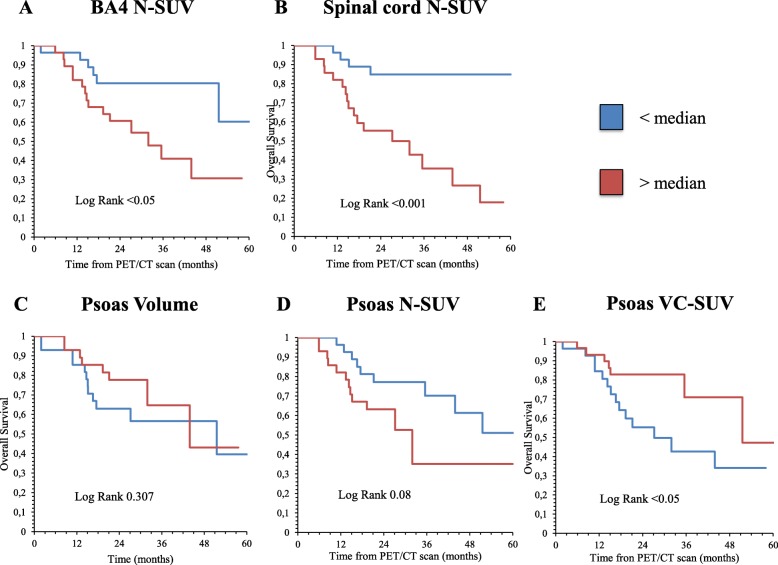
Metabolic predictors of patient outcome. Kaplan–Meier curves in ALS patients based on FDG PET/CT-derived parameters are represented. Long-term OS from the PET/CT scan time-point is stratified based on spinal cord N-SUV (**a**), BA4 N-SUV (**b**), Psoas volume (**c**), psoas N-SUV, and psoas VC-SUV (**e**). Each subgroup is divided according to the median

This difference was largely confirmed by the Kaplan–Meier analysis. ALS mortality rate was higher in patients with BA4 SUV lower than the median value (Fig. 4a). Conversely, N-SUV greater than the median value in the spinal cord predicted poorer overall survival (Fig. 4b). Shifting to the analysis of skeletal muscle, psoas volume was devoid of any predictive power (Fig. 4c). An increase in overall tracer retention, indexed by N-SUV, showed an appreciable, though not significant, association with a higher mortality rate (Fig. 4d), that was instead predicted by a heterogeneous tracer distribution in the muscle volume as defined by VC-SUV values over the median (Fig. 4e). These evaluations were confirmed by univariate analyses. However, the multivariate Cox regression model identified the spinal cord metabolic activation as the only independent predictive biomarker (hazard ratio 5.17, 95% confidence interval 1.74–15.38, *p* = 0.001), as detailed in Table 2.

**Table 2:** Predictive power of tested variables at univariate and multivariate analysis

Variable	Outcome results			Univariate analysis			Cox regression model		
	No. of patients	No. of deaths	Mortality rate (%)	Hazard ratio	95% confidence interval	*p*	Hazard ratio	95% confidence interval	*p*
Age									
< 63 years	28	7	25	1 (reference)	–	0.1			
> 63 years	28	14	50	2.37	0.771–7.32				
Psoas volume									
< 3.4	28	13	46	1 (reference)	–	0.17			
> 3.4	28	8	29	2.17	0.717–6.550				
Psoas N-SUV									
< 0.42	28	9	32	1 (reference)	–	0.41			
> 0.42	28	12	43	1.58	0.53–4.71				
BA4 SUV									
< 5.48	28	14	50	3.38	1.09–10.55	0.03			
> 5.48	28	7	25	1 (reference)	–				
ALSF score									
< 40	28	10	36	1 (reference)	–	0.57			
> 40	28	11	39	1.36	0.46–4.06				
Psoas CV-SUV									
< 6.59%	28	6	21	1 (reference)	–	0.016			
> 6.59%	28	15	54	4.23	1.31–13.62				
Spinal cord N-SUV									
< 0.92	28	4	14%	1 (reference)	–	0.001	1 (reference)	–	0.001
> 0.92	28	17	61	9.27	2.5–34.1		5.17	1.74–15.38	

## Discussion

The main finding of the present study is that the increase in FDG uptake caused by ALS in the spinal cord also involves the skeletal muscle. By contrast, this activation was not observed in the brain motor cortex that rather showed an opposite metabolic response characterized by a hypometabolism independent on the behavior of the spinal cord and skeletal muscle. The direct link between the metabolic pattern of the spinal cord and skeletal muscles selectively occurred in ALS patients and did not involve the striated myocardial muscle, independent on cholinergic neuromuscular synapses. In this series of patients, muscle volume was devoid of any prognostic predictive power that was instead marginally retained by FDG uptake and significantly predicted by its distribution within the tissue.

Although the exact mechanism underlying this metabolic response cannot be identified based on the present data, as demonstrated by the multivariate analysis, outcome prediction provided by the psoas metabolic pattern was largely dependent on the corresponding index in the spinal cord. This dependency suggests the presence of a common mechanism contributing to disease progression and indexed by the metabolic feature of both second motor neuron and its effector. By contrast, the absence of any prognostic implication for psoas volume, suggests that the prognostic capability of psoas metabolic activation reflects different mechanisms compared to the acknowledged link between muscle dimension and sarcopenia. Moreover, the psoas muscle size (largely superior to the PET scanner spatial resolution) allowed us to document significant heterogeneity of FDG uptake within this region. On the one side, this finding might suggest the presence of heterogeneous cell populations in each voxel within the analyzed muscular volume as a possible consequence of inflammatory infiltrates [20, 21]. Although no direct evidence is available to corroborate this hypothesis, this finding closely agrees with the evident role of inflammatory mechanisms in the progression of ALS muscular damage [22] both in patients [23] and experimental models [24]. On the other hand, this same heterogeneity also fits with the distribution of endoplasmic reticulum damage that has been observed at pathology both in ALS patients and in their experimental counterparts [25]. Current models do not consider any role for this organelle in glucose metabolism. However, our group recently reported evidence showing that FDG uptake strictly reflects the activation of NADPH generation by a pentose phosphate shunt selectively located within the endoplasmic reticulum in cancer cells [26, 27], cardiomyocytes [28], neurons [29] and, more importantly, in the skeletal muscle [30].

Unfortunately, the limited spatial resolution of PET imaging did not allow to evaluate uptake distribution within the spinal cord. Nevertheless, neuroinflammation is a key-signaling event in ALS [31]. Indeed, post-mortem neuropathological studies previously showed the presence of microglia and astrocytes activation, as well as lymphocytes and macrophages infiltrates in both motor cortex and spinal cord in ALS [32, 33]. These data suggest that activated microglia might accumulate within the degenerating areas propagating and sustaining tissue damage through the release of free radicals and other neurotoxic substances such as glutamate. According to this consideration, the divergent metabolic response of the brain cortex and spinal cord might reflect the contribution of different mechanisms or rather a different time sequence in first and second motor neuron ALS-related damage. In the latter hypothesis, the reduced FDG uptake of the motor cortex might be the consequence of its thinning [34] and the consequent underestimation related to the partial volume effect, rather than a true hypometabolism.

Several limitations of our study must be considered. First, brain and spinal cord/psoas muscle metabolic patterns of ALS patients were compared with normalcy databases collected from different cohorts. In healthy controls undergoing brain PET/CT this limitation was justified by the ethical concern of extending CT (increasing radiation exposure) to the whole body. The same consideration applies to the normal whole-body PET/CT scans, which were performed for oncological purposes and started from the orbit, normally excluding the brain. Second, the limited patient sample and the relatively large inclusion criteria did not allow us to describe the exact temporal progression between the brain and spinal cord/psoas muscle metabolic change. Similarly, the side coherence between brain metabolic impairment and psoas muscle hypermetabolism was not verified. Further studies, with larger sample sizes including ALS patients enrolled at different time points are needed to solve these issues.

## Conclusion

The analysis of psoas FDG uptake allowed us to identify a peculiar metabolic pattern of skeletal muscle in ALS, whose heterogeneity might provide prognostic insight in ALS clinical history. Of course, the differences in tracer retention of either psoas muscles or central nervous system are too small to consider a diagnostic or predictive capability for FDG imaging in ALS. Nevertheless, the similar metabolic activation of lower motor neuron and its effector seems to indicate that the selective response of these two regions might represent a potential target for patient characterization. From the clinical point of view, the availability of a new prognostic biomarker and its operator-independent nature could be invaluable for the development of new therapeutic approaches, especially in early phase clinical trials. On the pathophysiological ground, the observed interdependency with spinal cord metabolic pattern might suggest the existence of a common mechanism contributing to disease progression and indexed by the apparent increased FDG uptake in both second motor neuron and its effector.

## Supplementary information

**Additional file 1.** Regression between psoases average N-SUV and BA4 N-SUV.

## Data Availability

The datasets generated during and/or analyzed during the current study are available from the corresponding author on reasonable request.

## Abbreviations

AAC: Average attenuation coefficient
ALS: Amyotrophic lateral sclerosis
Bas: Brodmann areas
FDG: [18F]-fluorodeoxyglucose
HU: Hounsfield units
IBW: Ideal body weight
MNI: Montreal Neurological Institute;
N-SUV: Normalized average standardized uptake value
VC-SUV: Variation coefficient standardized uptake value
