# Supplementary figures and images for: Spinal cord hypermetabolism extends to skeletal muscle in amyotrophic lateral sclerosis: a computational approach to [18F]-fluorodeoxyglucose PET/CT images
Source: EJNMMI Res. 2020 Mar 23;10:23. doi: 10.1186/s13550-020-0607-5 (PMC7085992; doi:10.1186/s13550-020-0607-5)

**BA4 N-SUV**

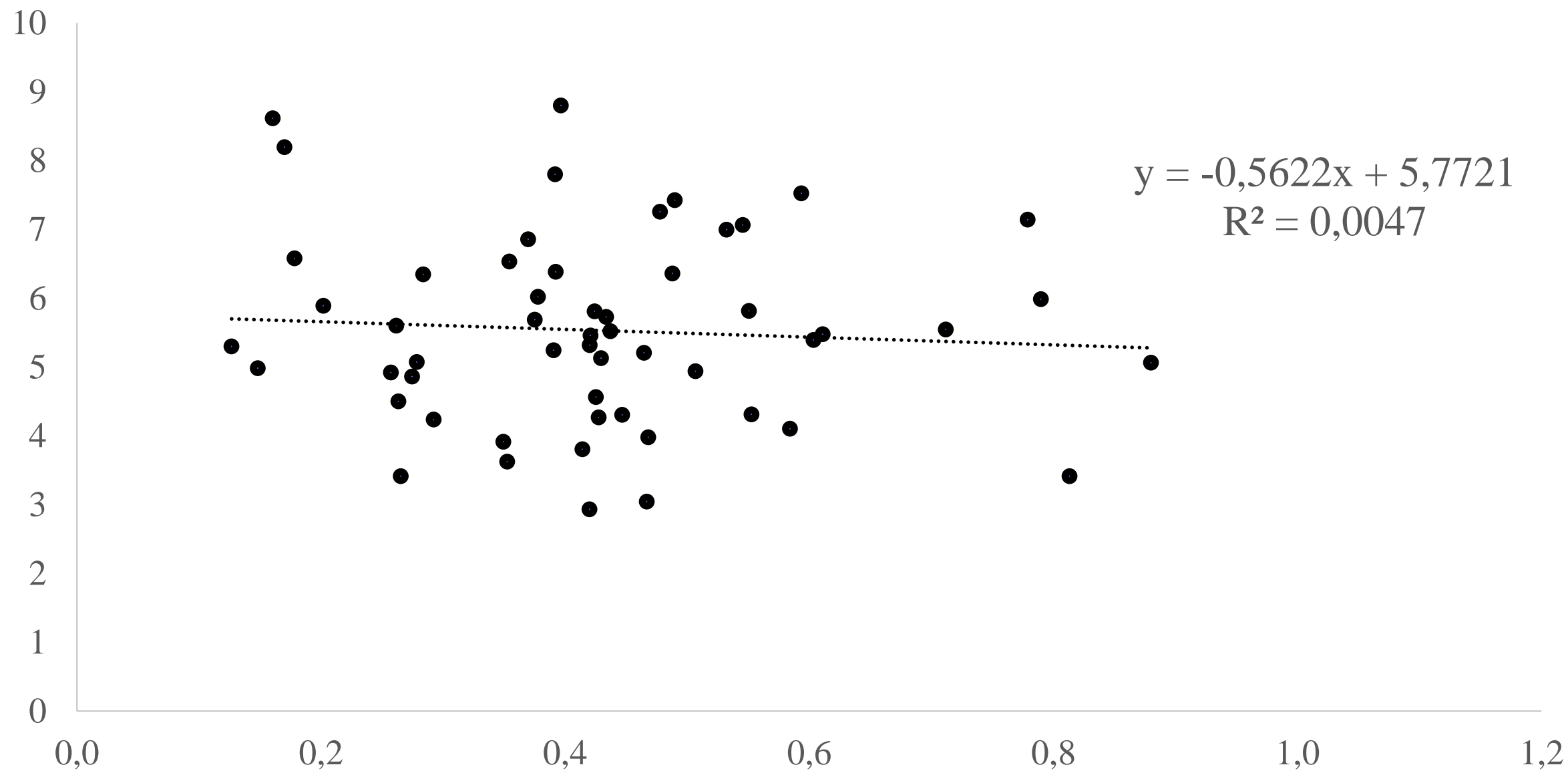

$y = -0,5622x + 5,7721$   
 $R^2 = 0,0047$

**Psoases average N-SUV**

Supplement: Supplementary file 1 — Additional file 1. Regression between psoases average N-SUV and BA4 N-SUV. [file 13550_2020_607_MOESM1_ESM.pdf]
